# Knowledge, attitude, and practice of waste handlers about medical waste management in Debre Markos town healthcare facilities, northwest Ethiopia

**DOI:** 10.1186/s13104-019-4174-7

**Published:** 2019-03-15

**Authors:** Teshiwal Deress, Mohabaw Jemal, Mekonnen Girma, Kasaw Adane

**Affiliations:** 1 Unit of Quality Assurance and Laboratory Management, School of Biomedical and Laboratory Sciences, College of Medicine and Health Sciences, University of Gondar, P.O. Box 196, Gondar, Ethiopia; 2 Microbiology Department, School of Biomedical and Laboratory Sciences, College of Medicine and Health Sciences, University of Gondar, P.O. Box 196, Gondar, Ethiopia

**Keywords:** Medical waste, Waste handler, Waste management, Knowledge, Attitude, Practice

## Abstract

**Objective:**

Medical waste is a total waste stream which is generated from the healthcare facilities during the healthcare delivery process. It can contain potentially hazardous substances for the human being and the environment. Waste handlers play a significant role for its proper management and they need to have adequate knowledge, attitude, and practices. The study aimed to evaluate the knowledge, attitude, and practices of waste handlers regarding medical waste management in Debre Markos town healthcare facilities, northwest Ethiopia.

**Results:**

A total of 55 medical waste handlers were studied from 12 healthcare facilities. Among this, 25 (45.4%) were diploma and certificate holders. The majority (69.1%) of the study participants were not provided with proper training. There was a lack of personal protective devices and waste management equipment supply. Regarding knowledge, attitude, and practices, 25 (45.5%), 43 (78.2%), and 44 (80%) of the study participants had adequate knowledge, favorable attitude, and adequate practice scores, respectively. There was high (30.9%) prevalence of needlestick and sharps injuries. Healthcare facilities should provide periodic training and adequate supplies for the waste handlers. Further study should be conducted on a large scale by including different levels of health facilities and regions of the country.

**Electronic supplementary material:**

The online version of this article (10.1186/s13104-019-4174-7) contains supplementary material, which is available to authorized users.

## Introduction

Medical waste is a total waste which is generated from the healthcare facilities (HCFs) during the course of the healthcare delivery process [1]. It includes syringes, needles, ampoules, dressings, disposable plastics and microbiological wastes [2]. The waste generated from the HCFs is broadly categorized as general and hazardous waste. According to the WHO estimation, the general and hazardous waste types constituted about 85% and 15%, respectively [3].

There are several terms used to represent wastes generated from the HCFs such as “medical waste”, “healthcare facility waste”, “biomedical waste”, “regulated medical waste” and “clinical waste” are used frequently in different articles. For this study, we used “medical waste” to represent the entire waste generated from the HCFs. Medical waste management is not well practiced throughout the world and very recently got its attention [4] due to increased awareness of HIV, HBV and HCV [5]. Medical waste can transmit more than 30 highly infectious bloodborne microorganisms [6].

Medical waste handlers (MWHs) play a key role in proper waste disposal as they are involved in the entire waste management processes. Optimum practice and use of personal protective measures depend on their level of knowledge and attitude about medical waste and its management. Medical waste management (MWM) process comprises interrelated key stages starting from segregation, collection, storage, transportation, treatment, and end up to its final disposal [7].

Appraisal of MWH’s knowledge and their skill for proper waste management could be a fruitful exercise to quantify and minimize occupational associated risks [7]. Waste handlers are often at high risk than healthcare professionals [3, 8]. Healthcare professionals once they produced the waste, they throw it in the garbage; however, waste handlers handle it extensively throughout and mostly very little attention is given for their safety. They have often observed washing medical devices and become at risk of cut with broken glassware and other sharp medical supplies [8].

Adequate knowledge, proper techniques, and safety practice measures can go a long way toward safe waste disposal and protection of the community from various adverse effects of hazardous waste [9]. Medical waste handlers are working in a very poor and unsafe working environment [10] and mostly they are victims of occupational health hazards from poor waste management practice. Adequate knowledge, favorable attitude, and adequate practices of waste handles are key factors for having proper hazardous MWM and to protect them from exposure to potentially hazardous substances [11]. Though proper MWM is an important area of public health particularly in developing countries including Ethiopia, there are very little related studies conducted among MWHs. In addition, from the empirical observation, MWHs among the Ethiopian HCFs are often practicing inappropriately. Therefore, the aim of this study was to evaluate the level of knowledge, attitude, and practices (KAP) among the MWHs towards proper waste management.

## Main text

### Methods

#### Study design and area

An institution based cross-sectional study was conducted from November 2016 to June 2017 in Debre Markos town HCFs. The town is found in Amhara regional state at 305 km far from Addis Ababa to the northwest. According to the Central Statistical Agency (CSA) report, the town has 119,000 population [12]. Currently, there are 12 clinics, 4 health centers, and 1 referral hospital in the town. The hospital has five wards with a total of 400 inpatient beds and currently it provides as a referral center for the primary hospitals and serve more than 5 million inhabitants in its catchment area [13]. Similarly, the primary health facilities (health centers and clinics) provide basic health services to the town and nearby areas. During the study period, there were a total of 55 female MWHs within the aforementioned health facilities.

#### Study population and sample size

Medical waste handlers were purposely selected because they are mostly involved in the handling and subsequent management of potentially hazardous medical wastes, cleaning contaminated medical equipment and HCF environments, and they become at high risk of hazardous substances. In addition, this group of healthcare workers plays a vital role in public health security in protecting the entire population. A total population sampling technique was employed to include all MWHs (55) from the 12 health facilities.

#### Data collection tools and procedure

A pre-tested interviewer-administered questionnaire and observational checklist were used to collect the data. The interviewer-administered data collection was used assuming waste handles might have limited educational level. Under this category, KAP domains were included which consisted of 21, 16 and 8 questions, respectively. Two types of observational checklists were also used to evaluate individual and HCFs waste management practices for which 9 and 7 questions were allocated, respectively. First, the data collection tool was prepared in English and then translated into the Amharic language. Before starting the actual data collection process, the tool was evaluated on similar study participants from other HCFs (hospital and health center) who were not included in the final study. Two data collectors (clinical nurse and medical laboratory technologist) have participated in the study and the medium of instruction was Amharic. After the completion of each study participant interview, the data collector has observed the study participant while he/she was doing the actual waste management practices using the study participant observation checklist. Then, the questionnaire and corresponding checklist were labeled using unique individual and HCF identification code numbers and then attached together. Finally, after completion of all the study participants’ evaluation, health facility observation was done.

#### Methods of scoring

Knowledge and practice responses were scored as either 1 or 0 points for correct and incorrect responses, respectively. Whereas, attitude questions responses were indicated with the three-point Likert type scale of measurement as “disagree”, “neutral” and “agree” and numerical values of 1, 2, and 3, respectively were given.

#### Data quality measures

Data collection tools were first prepared in English and then they were translated into the Amharic language. To assure the consistency of translation investigators were rechecked the translated questionnaire and corrective actions were as appropriate. Training was given for data collectors on how to approach and collect the data from the study participants. The pre-test was done on 10 waste handlers in other facilities. According to the pre-test results, a slight modification was done on the questionnaire and suggestions from different experts were included.

#### Data management and analysis

During the data collection process, questionnaires and observational checklists were checked for completeness. Then, data were entered into and analyzed using SPSS version 20 statistical software. Mean scores were calculated and used as a cut of a point to categorize KAP scores of the study participants as adequate knowledge, favorable attitude, and adequate practice scores. This was done by calculating mean scores of each domain which means composite scores under each domain were divided by the number of study participants. At that movement, scores below the mean scores were considered as inadequate knowledge, unfavorable attitude, and inadequate practice; whereas, score means and above were considered as adequate knowledge, favorable attitude, and adequate practices. In addition, descriptive statistics like proportion and frequency were computed as appropriate. Finally, the findings of this study were presented in the form of tables and texts as appropriate.

### Results

#### Socio-demographic and healthcare facility related characteristics

Fifty-five female MWHs were included in the study from 12 health facilities. Regarding their educational level, 25 (45.4%) of them were diploma and certificate holders and the remaining were primary school and below. With respect to previous training, only 17 (30.9%) of them were trained. Eleven (20%) and 22 (40%) of them were vaccinated for HBV and tetanus, respectively. Concerning the incidence of injuries, 17 (30.9%) of the study participants had encountered needlestick or sharp injury within a year preceding the data collection period (Table 1).

**Table 1:** Socio-demographic and HCF related factors of medical waste management at Debre Markos town HCFs, northwest Ethiopia, June 2017 (n = 55)

Socio demographic and HCF related variables	Variable category	Study participant n (%)
Working hours per day	< 8 h/day	8 (14.5)
	8 h/day	23 (41.8)
	> 8 h/day	24 (4 3.6)
Working experience	6–10	2 (3.6)
	1–5	49 (89.1)
	11–15	3 (5.5)
	> 15	1 (1.8)
Age group of the study participants (years)	21–25	29 (52.7)
	< 21	11 (20.0)
	26–30	8 (14.5)
	31–35	2 (3.6)
	> 40	5 (9.1)
Availability of personal protective equipment in the facility		
Duty Glove	Yes	52 (94.5)
	No	3 (5.5)
Duty boot	Yes	0
	No	55 (100)
Apron	Yes	48 (87.3)
	No	7 (12.7)

#### Knowledge, attitude, and practice

In this study, 25 (45.5%) of the study participants had adequate knowledge score. Only seven (12.7%) of the study participants were identified maximum storage time of hazardous MWs before treatment and/or disposal. Nineteen (34.5%) of the study participants identified the biohazards symbol. Concerning segregation of medical wastes, 40 (72.7%), 38 (69.1%), and 50 (90.9%) of the study participants identified that general, infectious and sharp wastes should be disposed of in a black, yellow and a safety box, respectively (Table 2). Forty-three (78.2%) of the study participants had a favorable attitude. The frequency of the study participants among each Likert type question is indicated in (Table 3). Regarding their practice, 44 (80.0%) of the study had adequate practice score and always used heavy-duty gloves as well. Forty-eight (87.3%) of them have been always used apron; however, none of them was used duty boots. The majority of the study participants 48 (87.3%), 50 (90.9%), and 49 (89.1%) used closed container while transport wastes, separately transport different waste types, and regularly clean reusable cleaning devices with disinfectant, respectively. However, only 24 (43.6%) used the trolley/wheelbarrow to transport waste containers to the treatment or disposal site.

**Table 2:** The frequency of study participants among each knowledge question at Debre Markos town HCFs, northwest Ethiopia, June 2017 (n = 55)

Variables	Response		
	Yes N (%)	No N (%)	Not sure N (%)
Does your facility generate MWs?	39 (70.9)	8 (14.5)	8 (14.5)
Do you know about medical waste management?	52 (94.5)	2 (3.6)	1 (1.8)
Is there any hazed associated with medical wastes?	54 (98.2)	0	1 (1.8)
Is needle-stick/sharp injury a concern?	54 (98.2)	1 (1.8)	0
Does wearing PPE reduce the risk of infection?	52 (94.5)	0	3 (5.5)
Are all medical wastes hazardous?	40 (72.7)	4 (7.3)	11 (20.0)
Do you know color coding segregation of medical wastes?	44 (80.0)	4 (7.3)	7 (12.7)
Should infectious waste containers be a label with biohazard symbol?	35 (63.6)	0	20 (36.4)
Should medical wastes be segregate at the source?	48 (87.3)	2 (3.6)	5 (9.1)
Does disinfection of medical wastes decrease infection transmission?	53 (96.4)	0	2 (3.6)
Do we need to close medical care waste containers while in transport?	50 (90.9)	2 (3.6)	3 (5.5)
Do we need to secure medical care wastes awaiting treatment/disposal?	50 (90.9)	1 (1.8)	4 (7.3)
Do you know about medical care waste disposal methods?	48 (87.3)	2 (3.6)	5 (9.1)

**Table 3:** Frequency distribution of study participants among each Likert item of medical waste management at Debre Markos town HCFs, northwest Ethiopia, 2017 (n = 55)

Predictor variables	Response options		
	Disagree n (%)	Neutral n (%)	Agree n (%)
Proper medical waste handling is an issue	6 (10.9)	1 (1.8)	48 (87.3)
Safe medical waste management need a teamwork	9 (16.4)	5 (9.1)	41 (74.5)
HIV can be transmitted through medical wastes	13 (23.6)	0	42 (76.4)
HBV can be transmitted through medical wastes	6 (10.9)	9 (16.4)	40 (72.7)
Medical wastes do not transmit any infectious diseases	10 (18.2)	1 (1.8)	44 (80.0)
Medical wastes should be segregated at the point of generation	4 (7.3)	2 (3.6)	49 (89.1)
Medical waste segregation can facilitate safe handling	5 (9.1)	1 (1.8)	49 (89.1)
Proper medical wastes disposal can prevent infection transmission	7 (12.7)	0	48 (87.3)
Medical waste disinfection can reduce the chance of contracting the infection	5 (9.1)	1 (1.8)	49 (89.1)
Wearing personal protective equipment help to reduce the risk of infection	5 (9.1)	0	50 (90.9)
Medical wastes management add the extra burden of work	8 (14.5)	1 (1.8)	46 (83.6)
Infectious medical wastes should be disinfected before disposal	9 (16.4)	3 (5.5)	43 (78.2)

#### Observational results

At the end of each working shift, waste handlers collected medical wastes from each service point and transport them to the treatment and/or disposal areas and they were immediately substituted with clean containers. Regarding PPE usage, 46 (83.7%) and 48 (87.3%) of the study participants used heavy-duty gloves and apron, respectively; however, none of them used duty. Seven (12.7%) study participants were observed using simple latex gloves and 1 (1.8%) used open shoes while they were cleaning medical devices and/or handling of hazardous medical wastes. Forty-seven (85.5%) of the study participants transported wastes as they were initially segregated. Only 32 (58.2%) of them used closed containers during transportation. Most (93%) HCFs used puncture-resistant containers to store hazardous wastes temporarily. Nine (75%) HCFs used incineration to treat all types of wastes and 3 (25%) HCFs used open burning only. Most HCFs have not specifically designed ash pit and they disposed of the ash in the latrine or open ground dumping.

### Discussion

Medical waste management has become a major problem for HCFs worldwide [14, 15]. The problem could be aggravated by the lack of adequate KAP and proper waste management utilities. Healthcare workers particularly waste handlers are mostly involved in its subsequent management and are potentially at risk [16].

Adequate training is a key factor for effective MWM [17, 18]; however, in this study, only 30.9% of the study participants were trained which is not in compliance with the national and international requirements [3, 19, 20]. This result was better than a finding from India in which none of the MWHs were trained at all [21]; however, better finding (81%) was obtained from Nigeria [22]. In addition, waste handlers are required to be vaccinated for HBV and tetanus [3, 23]; however, in this study, only 20% and 40% of them were vaccinated for HBV and tetanus toxoid, respectively. HBV vaccination in the current study was better than 0% and 9.1% findings from India [21, 24]. Concerning tetanus toxoid vaccination, the current result was also better than 26.7% of the Indian finding [21]. The possible explanation for this difference could be due to the difference in medical waste management between the two countries. In this study, a high number (30.9%) of the study participants have encountered needle-stick/sharp injury 12 months preceding the data collection period and it was supported with a study from eastern Ethiopia (30%) of the MWHs exposed to sharp injuries [10]. However, a better result was found from Gondar town in which 12.3% of the MWHs encountered needle-stick/sharp injuries [25]. This high proportion of needlestick/sharp injury incidence could be due to the lack of the proper PPE supply or lack of attention to the safety and security of the MWHs by the health facilities.

Adequate knowledge, favorable attitude, and adequate practice scores were found to be 45.5%, 78.2% and 80.0%, respectively and they were better than findings from India which were conducted in a different period and places of the country [26–28] and Bangladesh [29]. However, a better result was found from southern India, where 54% and 86.5% of the MWHs had adequate knowledge and a favorable attitude, respectively [30]. In the current study, only 80% of the study participants aware of color coding segregation. Specifically, 72.7%, 69.1% and 90.9% of them were able to identify that general, infectious and sharp wastes should be disposed of in a black, yellow and a safety box, respectively.

Medical waste handling is a hazardous activity and it needs the use of proper PPE; however, in this study, it was very poor. None of the study participants have used duty boots and 12.7% of them used simple latex gloves. This result was contradicted with the findings from Cameroon and Johor in which 100% of the MWHs used all the appropriate protective gears [31, 32] and the national regulations [19]. The possible explanation for this difference could be due to the lack of PPE supply and attention in the current study by the by the health facilities.

## Conclusion and recommendations

In this study, the level of knowledge, attitude, and practice scores were unsatisfactory and use of the appropriate personal protective devices and waste management utilities were limited. Healthcare facilities should provide periodic training and adequate supplies for the waste handlers. Further studies should be conducted at a large scale by including different levels of health facilities and regions in the country.

## Limitations

The study was conducted on a small number of study participants which may not represent other MWHs elsewhere in different regions and locations of the country.

## Additional file

**Additional file 1.** English version questionnaire.

## Abbreviations

DRERC: Departmental Research and Ethics Review Committee
HCF: health care facilities
KAP: knowledge, attitude, and practice
MWHs: medical waste handlers
MWM: medical waste management
PPE: personal protective equipment
SPSS: Statistical Package for Social Sciences
WHO: World Health Organization
