# Supplementary material for: Knowledge, attitude, and practice of waste handlers about medical waste management in Debre Markos town healthcare facilities, northwest Ethiopia
Source: BMC Res Notes. 2019 Mar 15;12:146. doi: 10.1186/s13104-019-4174-7 (PMC6420739; doi:10.1186/s13104-019-4174-7)
Supplement: Supplementary file 1 — Additional file 1. English version questionnaire. [file 13104_2019_4174_MOESM1_ESM.docx]

Healthcare facility identification code: _____

Study participant identification code: ______

## English Version Information Sheet

## Title of the Research: Knowledge, attitude, and practice of waste handlers about medical waste management in Debre Markos town healthcare facilities, northwest Ethiopia.

## Objective: To assess the knowledge, attitude, and practice of waste handlers about medical waste management in Debre Markos town healthcare facilities, northwest Ethiopia.

**Perceived benefits and risk:** You may not benefit personally; however, the results of this study may use to identify gaps and initiate responsible bodies to design and implement the appropriate strategy for the medical waste management. Being involved in this study does not induce any risk you will face.

**Confidentiality:** All personal identifiers and personal information will not be taken hence your responses will be kept confidential at all times. In addition, the information will be accessed by the researchers and research assistants only and the final data will be analyzed in aggregate.

**Participation and withdrawal**: Your participation in this research project which will take you about 12 minutes is fully voluntarily. You will be free to withdraw from the study at any time or not to answer any question if you want to do so.

**Persons to contact:** If you have any question/ concerns about this study, you can contact;

1. Mr**.** Teshiwal Deress (Mob: +251921638642, Email: [teshiwalderes@gmail.com](mailto:teshiwalderes@gmail.com))
2. Mr**.** Mohabaw Jemal (Mob: +251918723323, Email: [mohabawjemal@gmail.com](mailto:mohabawjemal@gmail.com))
3. Mr**.** Mekonnen Girma (Mob: +251918160169, Email: [mekonnen2302@gmail.com](mailto:mekonnen2302@gmail.com))
4. Mr**.** Kasaw Adane (Mob: +251919171986, Email: [kasawadane@gmail.com](mailto:kasawadane@gmail.com))

## English Version Consent Form

Dear participant you are among the study participants selected from the healthcare facility to provide your responses to this study. It is your full right to participate in the study and/ or not to answer any question if you do not want to do so. However, your honest responses to these questions will help us to get important data regarding knowledge, attitude, and practice of waste handlers towards medical waste management, so, you are kindly requested to take part in the study which will take you a maximum of 12 minutes to complete the interview. Would you be willing to participate, please?

1. Yes 2. No

***Dear interviewer!*** *For the sake of confidentiality please do not write the study participant’s name or other personal identifiers on the questionnaire!*

## English Version Questionnaire

***For the interviewer:*** *If the participant agrees to take part in the study, please read each instruction and question for her/him until he /she understand it well. If there is unclear instruction or question please elaborate it more without losing its original concept.*

**Section 1: Socio-demographic and health care facility related profiles**

Dear participant, please tell me your answer for the following questions among the given options I will read now.

| **No.** | **Socio-demographic and healthcare facility related variables** | **Answer** |
| --- | --- | --- |
|  | Sex? | 1. Male 2. Female |
|  | Age (in full years)? | _________ years |
|  | Religion? | 1. Orthodox Christian 2. Protestant Christian 3. Muslim 4. Other (specify) _______ |
|  | Marital status? | 1. Single 2. Married 3. Separated 4. Widowed |
|  | Educational level? | 1. Below primary 2. Primary 3. Secondary 4. Certificate 5. Diploma |
|  | In which health care facility are you working now? | 1. Hospital 2. Health center 3. Clinic |
|  | In which department/ section are you working now? **(More than one answers are possible)** | 1. OPD 2. Ward 3. Laboratory room 4. Emergency room 5. Others (specify) ______ |
|  | How much is your work experience as a healthcare facility waste handler? | __________Years |
|  | How much is your working hours per day as a healthcare facility waste handler? | __________Hours/day |
|  | Where do you get information about medical waste management? **(More than one answers are possible)** | Guideline  Training  Friend  Others (specify)_______ |
|  | Have you ever taken training on medical waste management or related issues? | 1. Yes 2. No |
|  | Have you taken the hepatitis B virus vaccine? | 1. Yes 2. No |
|  | Have you ever encountered any sharp /needlestick injury in the last 12 months? | - - - 1. Yes       2. No |
|  | Have you taken tetanus toxoid vaccine? | - - - 1. Yes       2. No |
|  | Which personal protective equipment is available in sufficient quantity in your facility? **(More than one answers are possible)** | - - - 1. Heavy-duty gloves       2. Heavy-duty boots       3. Apron       4. None of them is available       5. I am not sure |

**Section 2:** Questions to assess knowledge about medical waste management at Debre Markos town healthcare facilities.

***For the interviewer:*** *Please read each question and alternative options to the waste handler clearly and encircle the answer from the right margin of the table.*

| **No.** | **Questions** | **Answer** | | |
| --- | --- | --- | --- | --- |
|  |  | **Yes** | **No** | **Not sure** |
|  | Does the health facility in which you are working now generate biomedical wastes? | 1 | 2 | 3 |
|  | Do you know about medical waste management? | 1 | 2 | 3 |
|  | Is there any health hazard associated with medical wastes? | 1 | 2 | 3 |
|  | Is needle-stick or sharp injury a concern? | 1 | 2 | 3 |
|  | Does wearing personal protective equipment reduce the risk of infection? | 1 | 2 | 3 |
|  | Are all medical wastes biologically hazardous (infectious)? | 1 | 2 | 3 |
|  | Are all items potentially contaminated with body fluids considered as medical wastes? | 1 | 2 | 3 |
|  | Do you know about color coding segregation of medical wastes? | 1 | 2 | 3 |
|  | Do infectious waste containers be labeled with the biohazard symbol? | 1 | 2 | 3 |
|  | Should medical wastes be segregated into different categories at the point of generation? | 1 | 2 | 3 |
|  | Does the disinfection of medical wastes decrease the risk of infection transmission? | 1 | 2 | 3 |
|  | Do we need to close medical waste containers while transport? | 1 | 2 | 3 |
|  | Do we need to secure stored medical wastes waiting for treatment and/or disposal? | 1 | 2 | 3 |
|  | Do you know about medical waste disposal methods? | 1 | 2 | 3 |

1. What is the maximum time of infectious medical wastes can be stored before being treated or disposed of?
2. 24 hours
3. 48 hours
4. 72 hours
5. I don’t know
6. Which of the following is the internationally accepted symbol for the biohazard? (***dear interviewer please show the pictures to the study participant)***
   - - 1.
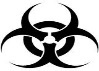

       2.
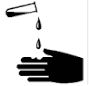

       3.
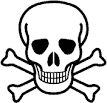

       4.
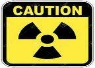

7. What type of medical waste should be disposed of in a yellow waste container (bin)?
8. General waste
9. Infectious waste
10. I don’t know
11. What type of medical waste should be disposed of in a black waste container (bin)?
12. General waste
13. Infectious waste
14. I don’t know
15. In which container medical supplies capable of causing puncture or cut be disposed?
    - - 1. Black waste bin
        2. Yellow bin
        3. Safety box
        4. I don’t know
16. How maximum full should be a safety box containing needle and/or sharp materials?
    - - 1. ½ full
        2. 3/4 full
        3. Full
        4. I don’t know
17. According to the World Health Organization (WHO) guideline, what is the maximum delay to start HIV post-exposure prophylaxis?
    - - 1. 24 hours
        2. 48 hours
        3. 72 hours
        4. I don’t know

**Section 3:** Questions to assess medical waste handlers’ attitude on medical waste management at Debre Markos town health care facilities.

***For the interviewer:*** *Please first read the following instruction clearly for the waste handler.*

*On the following scale of measurement 1-3 (1= Disagree (D); 2= Neutral (N), and 3= Agree (A); Please tell me your attitude regarding medical waste management for the statements I will read now.*

| **No.** | **What is your opinion/belief on the following statements?** | **D** | **N** | **A** |
| --- | --- | --- | --- | --- |
|  | Improperly managed medical wastes may cause infection. | 1 | 2 | 3 |
|  | Proper medical waste handling is an issue and a matter of concern. | 1 | 2 | 3 |
|  | Safe medical waste management is an issue involving the responsibilities of each healthcare staff. | 1 | 2 | 3 |
|  | HIV may be transmitted through medical wastes. | 1 | 2 | 3 |
|  | HIV post-exposure prophylaxis will help to prevent the development of HIV infection. | 1 | 2 | 3 |
|  | Hepatitis B virus may be transmitted through medical wastes. | 1 | 2 | 3 |
|  | Hepatitis C virus may be transmitted through medical wastes. | 1 | 2 | 3 |
|  | Medical wastes do not transmit any infection. | 1 | 2 | 3 |
|  | Medical wastes should be segregated into different categories at the point of generation. | 1 | 2 | 3 |
|  | Medical waste segregation facilitates safe handling of the waste. | 1 | 2 | 3 |
|  | Labeling medical waste containers do not add any value to waste management. | 1 | 2 | 3 |
|  | Proper medical waste disposal is important to prevent infection transmission. | 1 | 2 | 3 |
|  | Medical waste disinfection can reduce the chance of contracting the infection. | 1 | 2 | 3 |
|  | Wearing personal protective equipment helps to reduce the risk of infection. | 1 | 2 | 3 |
|  | Medical waste management adds the extra burden of work. | 1 | 2 | 3 |
|  | Medical waste management is only the responsibility of the institution. | 1 | 2 | 3 |
|  | Biohazardous wastes should be disinfected before disposal | 1 | 2 | 3 |

**Section 4:** Questions to assess practices of waste handlers about medical waste management at Debre Markos town health care facilities.

***For the Interviewer****: Please read the instruction, questions and their alternative options for the waste handler and then encircle their answer.*

Dear participant, please tell me your answer to the following questions and alternative options I will read now.

| **No.** | **Variables** | **Response options** |
| --- | --- | --- |
|  | How often do you use heavy-duty gloves while you clean or dispose of medical wastes? | Always  Sometimes  I don’t use |
|  | How often do you use duty boots while you clean or dispose of medical wastes? | Always  Sometimes  I don’t use |
|  | How often do you use an apron while you clean or dispose of medical wastes? | Always  Sometimes  I don’t use |
|  | Do you disinfect/decontaminate reusable cleaning devices after each use? | Yes  No |
|  | Do you collect infectious medical wastes from service area within 24 hours? | Yes  No |
|  | Do you always separately transport medical wastes according to segregation? | Yes  No |
|  | Do you always close medical waste containers during transport? | Yes  No |
|  | What kind of equipment do you use to transport medical wastes? **(More than one answers are possible)** | Trolley/wheelbarrow  Closed bucket  Open bucket  Other (specify)_____ |

**Dear participant thank you very much for your cooperation!**

Healthcare facility identification code: ____

Study participant identification code: _____

**Section 5: Study Participants’ Practice Observation Checklist**

**For the data collector:** Please observe the waste handler while she/he is on her/his duty. Based on your observation, please read each question and encircle the answer from the right margin of the table

| **No.** | **Variables** | **Response options** |
| --- | --- | --- |
|  | Which personal protective equipment is available in your facility? **(More than one answers are possible)** | - - - 1. Heavy-duty gloves       2. Boots       3. Apron       4. None is available |
|  | Did she/he use heavy-duty gloves? | - - - 1. Yes       2. No |
|  | Did she/he use boots? | - - - 1. Yes       2. No |
|  | Did she/he use an apron? | - - - 1. Yes       2. No |
|  | Did she/he disinfect/decontaminate reusable cleaning devices after use? | - - - 1. Yes       2. No |
|  | Did she/he collect infectious medical wastes from service area within 24 hours? | - - - 1. Yes       2. No |
|  | Did she/he transport medical wastes separately? | - - - 1. Yes       2. No |
|  | What is medical waste transporting equipment she/he used? **(More than one answers are possible)** | - - - 1. Trolley/wheelbarrow       2. Closed bucket       3. Open bucket       4. Other (specify)_____ |
|  | Does she/he close medical waste containers during transport? | - - - 1. Yes       2. No |

## Healthcare facility identification code: _____

## English Version Observational Checklist for the Healthcare Facilities

1. What medical waste storage method the facility used?
2. Onsite storage room
3. Puncture resistant storage containers
4. Other specify _________
5. Is there infectious waste stored for more than two days?
   - - 1. Yes 2. No
6. Does the facility use onsite medical waste treatment methods?
   - - 1. Yes 2. No **If No go to 606**
7. If yes on Qn no 3, what medical waste treatment method the facility used? **(Multiple answers are possible)**

Incineration

Sterilization

Chemical

Burning

Other (specify)_____

1. Is there an incinerator? **If no go to 607**

Yes

No

1. If yes, is it fenced to prevent unauthorized access?

Yes 2. No

1. What type of infectious waste disposal method used? **(Multiple answers are possible)**

Ash Pit

Needle pit

Pit burial

Other (specify)___
